# Gut Microbial Nitrate Reduction to Ammonia: A Possible Pathway of Biological Nitrogen Provisioning in Freshwater Insects

**DOI:** 10.1007/s00248-026-02771-w

**Published:** 2026-04-18

**Authors:** Kiera Nelson, Nick Peterson, Rozlyn Olson, Hanna Castillo, Charles Antwi, Stephanie Cromwell, M. Eric Benbow, Paul Ayayee

**Affiliations:** 1 Department of Biology, University of Nebraska at Omaha, Omaha, NE USA; 2 Department of Entomology, Michigan State University, East Lansing, MI USA; 3 Department of Osteopathic Medical Specialties, Michigan State University, East Lansing, MI USA; 4 Ecology, Evolution and Behavior Program, Michigan State University, East Lansing, MI USA; 5 AgBioResearch, Michigan State University, East Lansing, MI USA

**Keywords:** Nitrate reduction, Gut microbiome, Freshwater insects, Stable isotopes, Ammonium

## Abstract

Biologically provided nitrogen (from diet or symbionts) is overwhelmingly vital to animal life on Earth. Biological nitrogen provisioning (BNP), mainly via nitrogen fixation and recycling, is a well-established function of the gut microbiomes of various terrestrial insects but remains less well-studied in freshwater insects. One possible route for BNP in freshwater insects is microbial nitrate reduction to ammonium (NRA), which produces the same intermediate metabolite, ammonium, as microbial nitrogen fixation in terrestrial insects. In this study, we characterized gut microbiome composition using 16S amplicon sequencing, followed by compound-specific stable isotope analyses (CSIA), to examine gut microbial NRA as a possible function in nymphs of the herbivorous, filter-feeding mayfly (*Hexagenia bilineata*) and the predatory dragonfly (*Tetragoneuria spinigera)*. We first uncovered significant differences in microbiome community composition and elevated NRA potential in mayflies compared with dragonflies. We subsequently determined significantly greater ^15^N-signatures across essential amino acids (δ^15^N_EAA_) and non-essential amino acids (δ^15^N_NEAAs_) in the herbivorous mayfly than the predatory dragonfly, after incubation in K^15^NO_3_ for 5 days. This is interpreted as insect host uptake and incorporation of microbe-derived ^15^NH_4_ into amino acids following NRA. Taken together, the results presented here suggest that gut microbial NRA may be a potential mechanism for supplying biological nitrogen to freshwater insects, potentially helping alleviate nitrogen limitations. This work advances our understanding of freshwater insect host-gut microbe associations (nature and function) and the potential impacts of these associations on host and ecosystem processes.

**Supplementary Information:**

The online version contains supplementary material available at 10.1007/s00248-026-02771-w.

## Introduction

Extensive data exist on the occurrence and physiological relevance of biological nitrogen provisioning (BNP) in several terrestrial insects, such as cockroaches [1], termites [2–6], ants [7, 8], beetles [9–11]. Recent reviews summarizing the occurrence and physiological relevance of biological nitrogen provisioning (BNP), mainly through atmospheric nitrogen fixation, focused extensively on terrestrial insects [12, 13], with minimal mention of aquatic insects. This is despite established ecological concepts of trophic equivalences between freshwater insect functional feeding groups (FFGs) such as scrapers, filter-feeders, gatherer/collectors, omnivores, and predators, and terrestrial insect herbivores (phytophagous, xylophagous), predators, and omnivores, respectively [14–19]. As with terrestrial insects, aquatic insect FFGs tend to exhibit distinct gut microbial associations that, to some degree, reflect their trophic levels [20–25]. The distinct gut microbial associates of terrestrial insect functional feeding groups are proposed to be adaptive features that enhance their utilization of various diets and for diverse nutritional requirements [26–29]. Of these, biological nitrogen provisioning is crucial for amino acid synthesis and protein production by insect hosts.

Limited data exist on microbial nitrogen fixation in freshwater insects, although this process has been postulated for decades now [14, 18]. In addition to nitrogen fixation, no dataset exists on possible gut microbial provisioning of biological nitrogen to the host via nitrate reduction to ammonia (NRA) [30]. This is an entirely microbial process, as with nitrogen fixation, with no record to date of insects (terrestrial or freshwater) having this innate capability. Hypothetically, nitrate (NO^−^_3_) from water intake in the insect’s gut is converted to ammonium (NH_4_) by nitrate reductase enzymes [30, 31]. The generated ammonia/ammonium is a common intermediate between nitrate reduction and biological nitrogen fixation pathways, allowing host incorporation of the nitrogen via glutamine synthetase (GS) and glutamine-2-oxoglutarate amido transferase (GOGAT) pathways [30, 32].

A previous study using bulk stable isotope analysis found significantly higher δ^15^N signatures in tissues of small minnow mayflies (*Baetidae*) and net-spinning caddisflies (*Hydropsychidae*) following incubation in water containing a ^15^N-labeled nitrate,, relative to controls [33]. Although this study provided insights into the potential occurrence and relevance of this process in freshwater insects, several limitations emerged. Primarily, the use of bulk δ^15^N rather than compound-specific δ^15^N values of individual amino acids hindered any definitive determination of incorporated reduced nitrogen in amino acids extracted from host tissues. Furthermore, concerns about residual ^15^N-nitrate on insects after the initial experimental incubation period, due to inadequate washing or a subsequent incubation in normal water to ensure complete removal of ^15^N-nitrate, and lack of descriptions of associated gut microbiomes of the studied insects, limited the conclusions of that study.

In this study, we characterized the gut microbial composition and potential functions and explored gut microbial nitrate reduction to ammonia in nymphs of the herbivorous wiggler mayfly (*Hexagenia bilineata*: filter feeder) and the predatory Thunderbugs dragonfly (*Tetragoneuria spinigera)* as a potential route of biological nitrogen provisioning. This objective is based on the premise that these freshwater insects lack the metabolic capacity to reduce nitrate to ammonium on their own. To address concerns from the previous study, we used two insects with distinct feeding ecologies. Furthermore, we assessed nitrate reduction by quantifying ^15^N-nitrogen incorporated into amino acids extracted from host tissues using compound-specific stable isotope analysis (CSIA). This new analytical technique provides direct evidence for host uptake and utilization of microbe-provided nitrogen via nitrate reduction to amino acids. It is important to stress here that host uptake of the provided ^15^N tracer signal is only possible after gut microbial nitrate reduction to ammonium, or through other microbial processes that generate a microbial ^15^N metabolite and intermediary product reservoir for host assimilation. In addition, the study insects were maintained in water for 24 h after the incubation period to remove residual ^15^N-nitrate from the treatment insects and to minimize possible residual effects. Finally, we characterized the gut microbiomes of the two freshwater insects to assess whether they harbored distinct microbiomes with distinct potential functions relevant to nitrate reduction.

Overall, we anticipated differences in gut microbiome composition and potential functions between the filter-feeding mayfly and the predatory dragonfly, reflecting the role of diet (and possibly trophic level) on shaping these associations. However, we do not anticipate differences in the gut microbiomes of the control (incubated in normal ^14^N-nitrate water) and treatment (incubated in ^15^N-nitrate water) groups of each species, since the nitrate concentrations used in this study were below host-lethal limits and were not expected to adversely affect gut microbes. We also predicted higher microbe-mediated nitrate reduction to ammonium (elevated ^15^N signatures in amino acids) in the herbivorous mayfly relative to the predatory dragonfly, given the higher nitrogen limitation of herbivores, the associated gut microbiome, and the inability of insects to carry out this metabolic process.

## Materials and Methods

### Aquatic Insects and Experimental Design

Immature nymphs of the mayfly (*Hexagenia bilineata)* (250 individuals) and dragonfly (*Tetragoneuria spinigera*) (250 individuals) were obtained from a small company that rears them as fishing bait (Thereelthingbait, Green Bay, WI, http://www.thereelthingbait.com/index.htm). The objective was to broadly assess this potential gut microbial function across aquatic stages (immature stages, regardless of nymphal age) of the two species in this study, since their ecologies differ from those of adults. Thus, we did not factor in age-specific information on the nymphs acquired, as little is known about how the developmental stage affects the gut microbiota and the isotopic incorporation of freshwater insects. Upon receipt, insects were sorted into control and treatment groups by species, placed in containers with artificial stream water (ASW) [34] with a water pump for aeration. For the treatment incubation solution, approximately 0.01 g of K^15^NO_3_ (98 atom‰ ^15^N, 2 atom‰ ^14^N) was added to 1 L of ASW to a final concentration of 0.0989 mM (10 mg/L). The control solution was prepared by adding 0.01 g of K^14^NO_3_ (99.6 atom‰ ^14^N, 0.4 atom‰ ^15^N) to 1 L of ASW to achieve the same final concentration (0.1 mM). The rationale for using K^15^NO_3_ is to enable isotopic tracing of the provided inorganic ^15^N into organic compounds (amino acids) following microbe-mediated nitrate reduction, which can then be quantified via compound-specific isotope analysis (CSIA).

**Fig. 1:**
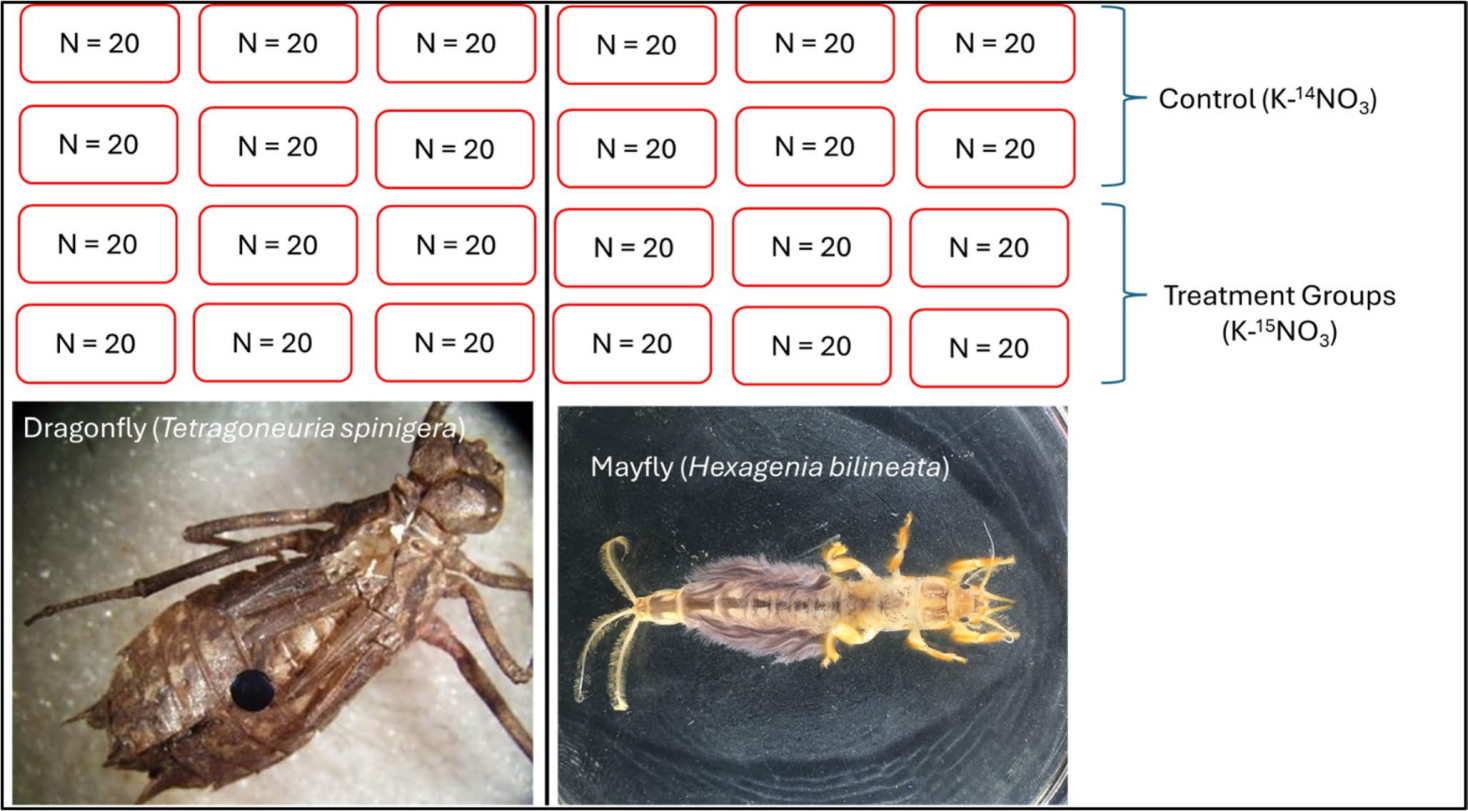
The experimental design of the study. Approximately 4th instar nymphs of the herbivorous wiggler mayfly (*Hexagenia bilineata*: filter feeder) and the predatory Thunderbugs dragonfly (*Tetragoneuria spinigera*) were maintained in six replicate containers for control (normal, ^14^N-Potassium Nitrate, K-^14^NO_3_) and treatment (^15^N-labeled Potassium Nitrate, K-^15^NO_3_) groups for 5 days. The minimum requirement was 20 insects per container. However, numbers ranged from 20 to 22 in some cases

To eliminate and minimize concerns about bacterial contamination, the ASW solution was filtered through a 0.2 μm membrane filter (EMD Millipore, Billerica, MA, USA), which removes particles larger than 0.2 μm (most bacteria). The selected concentrations are within the tolerance limit for various freshwater insects [35, 36]. Insects were incubated in containers with 250–350 ml of control and experimental ASW for 5 days, and in normal ASW for 24 h at the end of the incubation period. The 5-day incubation period was selected based on a combination of the previously reported 3-day incubation period for mayflies [33] and daily mortality monitoring. Once mortality was observed in groups (control and treatment), by day 5, the study was concluded. For each species, there were six replicate containers, with at least 20 insects per container in the control and treatment groups (*n* = 20 nymphs per container, *N* = 6 containers per species for control and treatment groups, total of 24 containers) (Fig. 1). Although we had a minimum requirement of 20 insects per container numbers ranged from 20 to 22 across in some cases.

### Sample Processing for Microbiome Characterization and Stable Isotope Analyses

Briefly, after a 24-hour wash period in nanopore water to remove residual/attached K-^15^NO^−^_3_ or K-^14^NO^−^_3_ at the end of the incubation period, all insects from all 24 containers were surface-sterilized by serially washing for 1 min in a 10% bleach solution, 10% detergent, and nanopore water. The digestive tracts of control insects were removed first, followed by the treatment samples to minimize transfer of ^15^NO^−^_3_ between treatment and control groups [37]. Extracted DNA was stored at −20 °C till sequencing. The remains (head capsule and carcass) were rinsed once again and stored in −80 °C for stable isotope analyses.

#### Sample Selection for Microbiome Characterization

Because we could not sequence the microbiomes of all 120 insects in each category (mayfly_^14^N, mayfly_^15^N, dragofly_^14^N, dragonfly_^15^N), making a total of 480 individual insect samples (Fig. 1), we randomly pooled extracted gut samples together from each category to generate a total of 90 samples (mayfly^14^N, *n* = 21; mayfly_^15^N, *n* = 25; dragonfly_^14^N, *n* = 22; and dragonfly_^15^N, *n* = 22) for DNA extraction. These samples were submitted for library prep and 16 S rDNA amplicon sequencing at the UNMC Genomics Core Facility. Library prep and sequencing of the approximately 150 bp V4 hypervariable region of the 16 S rDNA gene were carried out on the Illumina NovaSeq 500/550 platform using established, previously reported protocols [21, 22].

#### Amplicon Data Processing and Analyses

Standard read-quality processing was carried out in Nephele [38] and DADA2 (V. 1.30) in R [39] for comparative purposes, truncating at ~ 130 base pairs, after removing the first 20 base pairs of each fragment, and with an error cut-off of 2. Subsequently, reads were merged, chimeras were removed, ASVs were generated, and ASV taxonomies were assigned using the SILVA 138.1 16S rRNA gene reference database [40]. A classical ASV table was generated and used for downstream analyses in QIIME v.1.8 [41, 42] and MicrobiomeAnalyst [43] for comparative purposes. In both instances, the ASV table was curated by removing unclassified bacteria, mitochondria, chloroplasts, and eukaryotic reads prior to analysis, and the analyses followed standard practices. Three samples with too few reads were removed for further analysis, resulting in a final ASV table with 87 samples. This dataset was rarefied to 5,798 reads per sample for microbial diversity (alpha and beta) analyses [44, 45]. Rarefaction was chosen as part of the analytical process given its general consensus as a standard practice [46, 47]. Shannon’s evenness [48] was estimated to assess the evenness of the gut microbiome, and significant differences among the comparative groups (mayfly_^14^N, mayfly_^15^N, dragonfly_^14^N, and dragonfly_^15^N) were assessed using the nonparametric Wilcoxon test. The Shannon index was selected because, in addition to providing a measure of the evenness of microbial taxa across samples, it is also highly sensitive to the addition or loss of rare species, which is relevant to the biological function of interest (nitrate reduction to ammonium) in this study. To assess compositional information (beta diversity), a Bray–Curtis dissimilarity distance matrix [49, 50] was generated using the rarefied table. Differences among these categorical groupings were assessed using permutational multivariate analysis of variance (PERMANOVA [51] based on the Bray-Curtis distance, followed by pairwise comparisons. To identify bacterial taxa that underline differences in composition among experimental groups, the group_significance command in QIIME was used to identify differentially abundant taxa. This produces a table of the associated Kruskal–Wallis test statistic and p-value for each ASV, along with the calculated abundance scores across experimental groups. Relative abundance calculations enable quantitative comparisons of ASVs with differential abundance (plotted at the family level for a clearer, less noisy overview of taxa that differ among experimental groups). Inferred potential microbiome functional capabilities were assessed using FAPROTAX [52]. This allows estimation of inferred microbial metabolic capabilities/functions based on the taxonomic composition of the ASVs in the rarefied ASV table used for analyses. The group_significance command in QIIME was used to identify differentially abundant potential microbial functions, and their relative abundances were calculated for visualization, as with taxa above. Raw reads have been deposited into the Sequence Read Archive database (BioProject Number: PRJNA1396918).

#### Sample Selection for Compound-Specific Stable Isotope Analyses (CSIA)

All samples were freeze-dried under vacuum at −120 °C for 48 h, then milled in a ball mill. Similarly, because we could not analyze all 480 individual insects across the four categories (mayfly_^14^N, mayfly_^15^N, dragonfly_^14^N, and dragonfly_^15^N) via CSIA (Fig. 1), replicate lyophilized samples from the same experimental groups (control or treatment for both species) were pooled to obtain sufficient material (0.25 g) from each category to generate a total of 18 samples (^14^N_mayflies, *n* = 4; ^15^N_mayflies, *n* = 5; ^14^N_dragonflies, *n* = 4; and ^15^N_dragonflies, *n* = 5). Samples were placed in tin capsules, folded into balls, and submitted to the stable isotope facility at the University of California, Santa Cruz (https://isotope.ucsc.edu/analysis) for compound-specific stable isotope analysis (CSIA).

#### Compound-Specific Stable Isotope Analyses (CSIA)

Hydrolysis and derivatization of samples were performed at the USSC using established methods [53, 54]. Dried samples were hydrolyzed under standard conditions (6 N HCl for 20 h at 110 °C), and the resulting hydrolysate purified using cation exchange chromatography (Dowex 50WX8-400 ion exchange resin [55]. Isopropyl-TFA derivatives were prepared using the Silfer method [56] and further purified by solvent extraction [57]. Derivatized samples were analyzed using a Thermo Trace 1310 gas chromatograph coupled to an Isolink II (Thermo combustion reactor, 1000 °C)/Conflo IV and a Thermo Delta V Plus isotope ratio mass spectrometer. Amino acids were separated for δ^15^N analyses using a BPX5 column (60 m×0.32 mm, 1 μm film thickness; SGE Analytical Science, Trajan, Austin, TX, USA). The injector temperature was 250 °C with a split He flow rate of 2 mL/min. The GC temperature program for nitrogen isotope analysis was: initial temp = 70 °C hold for 1 min; ramp 1 = 10 °C/min to 185 °C, hold for 2 min; ramp 2 = 2 °C/min to 200 °C, hold for 10 min; ramp 3 = 30 °C/min to 300 °C, hold for 6 min. With this analytical approach, δ^15^N values was reproducibly measured for alanine (Ala), aspartic acid + asparagine (Asp), glutamic acid + glutamine (Glu), leucine (Leu), isoleucine (Ile), proline (Pro), valine (Val), glycine (Gly), lysine (Lys), serine (Ser), phenylalanine (Phe), threonine (Thr), and tyrosine (Tyr). Reproducibility for these amino acids is typically less than 1‰. The directly measured amino acid δ^15^N values were corrected using bracketing external standards [54].

#### Stable Isotope Data Processing and Analyses

We used the standard least squares model with the respective essential amino acid (δ^15^N_EAA_) and non-essential amino acid (δ^15^N_NEAA_) values as the dependent variables, and experimental groups (mayfly_^14^N, mayfly_^15^N, dragonfly_^14^N, and dragonfly_^15^N), and the respective amino acids - seven essential amino acids and six non-essential amino acids-, and their interactions as the model effects. This was followed by normalizing all treatment EAAs and NEAAs to their corresponding control EAAs and NEAAs to determine the offsets between control and experimental values across individual amino acids (Δ^15^N _Control EAA_ = (δ^15^N_Control EAA_ – δ^15^N _Treatment EAA_). The same mixed-model analysis with the offset values (Δ^15^N _Control EAA_) as the dependent variable was performed. All statistical analyses were performed in JMP 18 (JMP, SAS Inc. NC, USA).

## Results

### Gut Microbiome Characterization

**Fig. 2:**
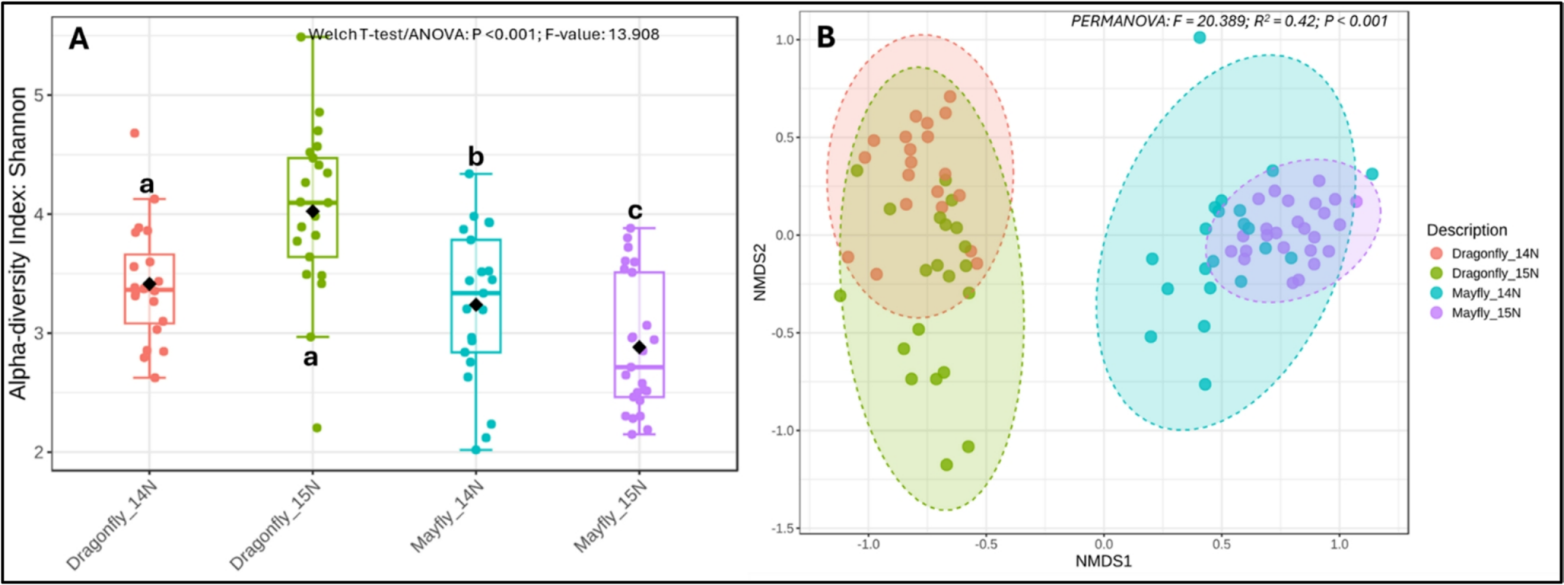
(**A**) Microbiome evenness and (**B**) NMDS showing community composition across control (mayfly_^14^N and dragonfly_^14^N) and treatment (mayfly_^15^N and dragonfly_^15^N) insects studied. Community composition was similar among controls and treatment groups of each species. Different letters in (**A**) indicate statistically significant differences following pairwise comparisons (*P* = 0.05). Microbiome community composition in (**B**) was significant between mayflies and dragonflies (both controls and treatments) at *P* = 0.05. NMDS stress = 0.13

Across the 87 samples, we retained 96.2% of input reads and recovered 4,389 ASVs. The rarefaction depth (5,798 reads per sample) adequately captured diversity among samples (Fig. S1). Evenness analyses at the 5,798-rarefaction limit determined significant differences between the control and treatment groups of both mayfly and dragonfly species (Fig. 2A). The mayfly_^14^N and dragonfly_^14^N evenness were comparable. However, the evenness in the mayfly_^15^N samples was significantly lower than that in the corresponding mayfly_^14^N samples. In contrast, the evenness of the dragonfly_^15^N was significantly higher than that of the control samples. The two treatment groups also differed, with dragonfly_^15^N exhibiting higher evenness than the mayfly_^15^N samples, suggestive of species-level differences (Fig. 2B) (Table S1B). Support for this possibility is in the determination that overall, mayflies had significantly different microbiome composition than the dragonflies (PERMANOVA: F-value = 20.389; R^2^ = 0.42; P-value = 0.001) (Fig. 2B) (Table S1B). However, we also uncovered significant within-species compositional differences in both species (Table S1B).

**Fig. 3:**
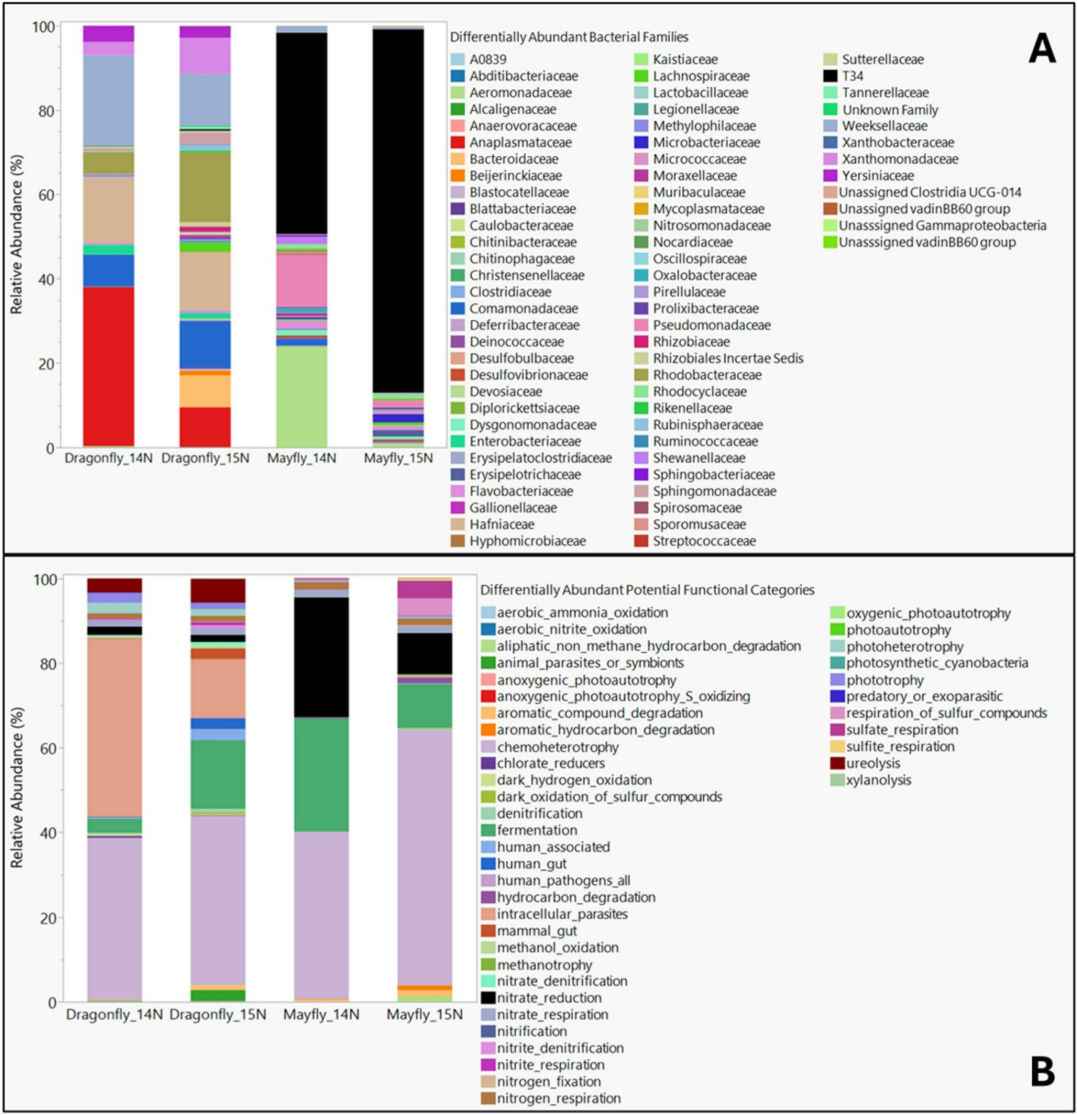
(**A**) The relative abundances of 72 differentially abundant bacterial ASVs (at the family level) out of a total of 4,389 ASVs (Kruskal–Wallis; FDR-adjusted P-value = 0.05), likely driving differences in determined microbial community composition between mayflies and dragonflies. (**B**) The relative abundances of 41 differentially abundant potential bacterial metabolic functional groups out of 91 available functional groups following FAPROTAX analysis (Kruskal–Wallis; FDR-adjusted P-value = 0.05), highlighting distinct functional potentials between mayflies and dragonflies

We subsequently identified 72 bacterial families through differential abundance analyses, representing microbiome composition differences of mayflies and dragonflies (Fig. 3A). There was notably lower taxonomic resolution at the genus level, resulting in more Unassigned ASVs at the genus level (22 unassigned genus-level taxa) (Fig. S2), compared to the family level (4 unassigned family-level taxa). We opted to describe the community composition at this level, due to the fewer unassigned designations. Of these, the nitrate-reducing Burkholderales family T34, *Pseudomonadaceae*, *Aeromonadaceae*,* Dysgomonadaceae*, *Flavobacteriaceae*, *Microbacteriaceae*, *Micrococcaceae*, *Rhodocyclaceae*, and *Shewanellaceae* were more abundant in mayflies relative to dragonflies (Fig. 3A) (Table S2A). In contrast, *Anaplasmataceae*,* Bacteroidaceae*,* Beijerinckiaceae*,* Comamonadaceae*,* Enterobacteriaceae*,* Hafniaceae*,* Lachniospiraceae*,* Rhozobiaceae*,* Rhodobacteraceae*,* Rikenellaceae*,* Rubinisphaeraceae*,* Sphingomonadaceae*,* Weeksellaceae*,* Xanthomonadaceae*, and *Yersiniaceae* were more abundant in dragonflies (Fig. 3A) (Table S2A). Within-species variations in microbiome composition between the control and treatment mayfly and dragonfly samples were observed, but overall, between-species differences are more notable and relevant in this study (Fig. 3A) (Table S2A)**.**

Identified taxa driving the compositional differences among control and treatment mayfly and dragonfly groups were further underscored by differences in potential functions. We identified 41 potential microbial functional pathways/categories that differed among mayfly and dragonfly samples (Fig. 3B) (Table S2B). Of these, Denitrification, hydrocarbon degradation, nitrate reduction, nitrate respiration, respiration of sulfur compounds, sulphate respiration, and sulfite respiration were potential microbial functional pathways elevated in mayfly samples compared to dragonflies (Fig. 3B). In contrast, human (pathogen, gut, and associated microbes), animal intracellular parasitic, methanotrophy, nitrogen respiration, photoheterotrophy, phototrophy, and ureolysis microbial functionalities were higher in dragonfly samples (Fig. 3B). Notably, potential nitrate reduction functionalities were higher in the mayflies (both mayfly_^14^N and mayfly_^15^N) compared to dragonflies.

### Gut Microbiome Nitrate Reduction

Overall, we obtained high-quality δ^15^N_AA_ data for six non-essential amino acids (NEAA) - Alanine (Ala), Aspartate (Asp), Glutamate/Glutamine (Glu), Glycine (Gly), and Serine (Ser)-, and six essential amino acids (EAA) – Isoleucine (Ile), Leucine (Leu), Lysine (Lys), Phenylalanine (Phe), Threonine (Thr), Valine (Val), and one conditionally-essential amino acid, Tyrosine (Tyr) (Table 1).

**Table 1:** Raw δ^15^N_AA_ data for seven essential amino acids (EAAs) and six non-essential amino acids (NEAAs) quantified following compound-specific stable isotope analyses of control and treatment mayfly and dragonfly samples (*N* = 18 in total). NEAAs: Ala, Gly, Ser, Pro, Asp, and Glu. EAAs: Thr, Val, Leu, Ile, Phe, Tyr, and Lys

Groups	Ala	Gly	Ser	Pro	Asp	Glu	Thr	Val	Leu	Ile	Phe	Tyr	Lys
Mayfly_^14^N	12.7	3.3	4.8	14.3	12.6	14.6	−4.3	10.5	5.1	8.0	4.3	3.6	2.5
Mayfly_^14^N	13.6	2.7	4.9	16.7	14.0	15.3	−4.1	11.1	6.3	8.9	3.4	3.9	2.7
Mayfly_^14^N	13.5	2.5	4.2	16.6	13.1	14.9	−4.5	10.2	5.7	8.4	2.7	3.7	2.4
Mayfly_^14^N	7.8	2.5	2.2	11.1	8.8	10.6	−11.7	12.2	10.3	14.7	1.2	−0.8	0.4
Mayfly_^15^N	17.4	3.9	4.9	23.6	15.6	24.9	−4.8	12.1	4.1	8.9	3.1	6.2	2.7
Mayfly_^15^N	19.1	5.4	5.8	25.4	16.6	25.4	−4.1	12.3	3.7	9.1	2.7	6.3	2.9
Mayfly_^15^N	36.6	7.2	10.1	39.1	37.2	79.1	−3.4	15.3	8.7	12.9	4.7	14.5	3.5
Mayfly_^15^N	30.2	7.3	7.9	48.1	29.4	71.6	−5.1	16.1	8.2	14.7	4.3	12.0	3.2
Mayfly_^15^N	53.5	11.3	11.4	66.7	43.9	93.3	−3.6	17.7	10.0	15.1	5.5	9.9	3.3
Dragonfly_^14^N	9.1	2.1	2.9	10.8	9.1	12.2	−10.3	11.9	9.3	14.9	2.3	0.3	0.9
Dragonfly_^14^N	9.3	3.3	3.3	11.1	10.1	12.5	−9.6	12.5	10.2	15.4	2.3	−0.1	1.1
Dragonfly_^14^N	13.3	4.9	4.8	16.3	14.5	15.5	−4.3	10.9	6.4	8.9	2.7	3.9	2.4
Dragonfly_^14^N	8.9	1.8	1.9	11.2	9.1	11.5	−11.4	11.8	9.4	15.1	1.3	−0.1	0.8
Dragonfly_^15^N	9.3	2.5	2.9	11.9	8.5	12.6	−11.4	13.0	10.6	17.3	1.3	−0.3	1.9
Dragonfly_^15^N	8.4	2.6	2.4	10.2	8.0	11.7	−11.6	11.7	9.1	14.9	0.4	−0.2	1.2
Dragonfly_^15^N	10.4	3.6	3.2	11.6	8.1	13.1	−9.7	12.9	10.6	16.5	2.0	0.5	2.2
Dragonfly_^15^N	18.7	2.4	2.9	10.7	10.0	39.1	−11.5	11.7	8.4	14.5	1.4	−0.7	0.7
Dragonfly_^15^N	10.1	3.9	3.2	11.1	9.6	14.8	−9.9	12.8	10.1	15.9	1.7	0.6	1.7

**Fig. 4:**
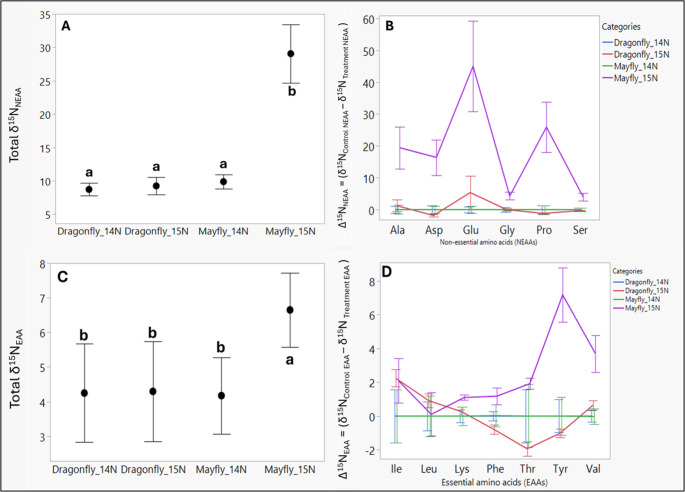
Compound-specific stable isotope (CSIA) results. (**A**) Combined 15 N isotope values across all six non-essential amino acids (NEAAs)(Total δ^15^N_NEAA_) extracted and analyzed from control and treatment mayfly and dragonfly experimental groups, and (**B**) the ^15^ N isotope enrichment or depletion across all six NEAAs from treatment groups (mayfly_^15^N and dragonfly_^15^N) relative to the respective control samples means (Δ^15^N _NEAA_). (**C**) Combined ^15^ N isotope values across all seven essential amino acids (EAAs)(Total δ^15^N_EAA_) extracted and analyzed from control and treatment mayfly and dragonfly experimental groups after, and (**D**) the ^15^ N isotope enrichment or depletion across all seven EAAs from treatment groups (mayfly_^15^N and dragonfly_^15^N) relative to the respective control samples means (Δ^15^N_EAA_). Shown are the mean values and the standard error of the mean in all panels. Nonessential amino acids were Ala, Asp, Glu, Gly, Pro, and Ser. Essential amino acids were Leu, Ile, Lys, Phe, Thr, and Val, with the conditionally essential Tyr

For non-essential amino acids (NEAAs), there was an overall significant model effect (F_(23, 107)_ = 9.61; *P* < 0.001), with significant effect of the experimental groups (F_(3, 104)_ = 31.22; *P* < 0.001), amino acids (F_(5, 102)_ = 14.6; *P* < 0.001), and their interaction (F_(15, 92)_ = 3.04; *P* < 0.001). Pairwise comparisons revealed the mayfly_^15^N samples had the highest δ^15^N_NEAA_ (29.1 ± 1.94, mean ± S.E) relative to the other samples (Fig. 4A), followed by the mayfly_^14^N (9.9 ± 1.74), the dragonflies_^15^N (9.2 ± 1.74), and the dragonflies_^14^N (8.73 ± 1.74). The ^15^N-enrichment in the six NEAAs across the ^15^N_mayflies suggests host utilization of microbe-derived ^15^NH_4_ from tracer nitrate (K-^15^NO_3_), either following NRA reduction or other microbial processes in the gut. In contrast, the mean values of dragonfly_^15^N and their corresponding control samples did not differ significantly. The lack of ^15^N-enrichment in the control groups of both species is expected, as these groups were not exposed to K-^15^NO_3_. Calculated offsets (Δ^15^N_NEAA_ = (δ^15^N_Control NEAA_ – δ^15^N _Treatment NEAA_) across the six NEAAs between the treatment and control samples similarly had an overall significant model effect (F_(23, 107)_ = 6.65; *P* < 0.001), and significant group (F_(3, 104)_ = 28.73; *P* < 0.001), amino acids (F_(5, 102)_ = 3.65; *P* < 0.001), and interactive (F_(15, 92)_ = 2.92; *P* < 0.001) results. Individual NEAA offsets (Δ^15^N_NEAA_) across all sample groups demonstrate substantial ^15^N-enrichments greater than 10 (> 10) across four NEAAs (Ala, Asp, Glu, and Pro), and around 5 for the remaining two (Ser and Gly) (Fig. 4B).

For essential amino acids (EAAs), there was an overall significant model effect (F_(27, 125)_ = 60.0; *P* < 0.001), with significant effect of the experimental groups (F_(3, 122)_ = 13.04; *P* < 0.001), amino acids (F_(6, 119)_ = 238.40; *P* < 0.001), and their interaction (F_(18, 107)_ = 6.50; *P* < 0.001). Pairwise comparisons show that mayfly_^15^N had the highest δ^15^N_NEAA_ value (6.65 ± 0.33, mean ± S.E) relative to other samples (Fig. 4C), followed by dragonfly_^15^N (4.30 ± 0.33), dragonfly_^14^N (4.25 ± 0.37), and mayfly_^14^N (4.17 ± 0.36). Similarly, the ^15^N-enrichment in the seven EAAs across the mayfly_^15^N may reflect host utilization following NRA reduction or other microbial processes in the gut. Similarly, for EAAs, the mean values of dragonfly_^15^N and their corresponding control samples did not differ significantly. Calculated offsets (Δ^15^N_EAA_ = (δ^15^N_Control EAA_ – δ^15^N _Treatment EAA_) across the seven EAAs between the treatment and control samples similarly had an overall significant model effect (F_(27, 127)_ = 14; *P* < 0.001), group (F_(3, 124)_ = 13.45; *P* < 0.001), and interaction effects (F_(18, 107)_ = 2.92; *P* < 0.001). Individual EAA offsets (Δ^15^N_EAA_) across all sample groups show lower ^15^N-enrichments (~ 2 or lower) for five EAA s (Ile, Leu, Lys, Phe, and Thr), and two with enrichments above 4 (Tyr and Val) (Fig. 4D).

## Discussion

Nitrate reduction to ammonium (NRA) is presently understood to be a strictly microbial process [30, 31, 58]. Thus, the guiding conceptual framework of this study is based on the understanding that insects are incapable of this process and that stable isotope analytical methods like CSIA, rather than gene expression methods (RT-qPCR or RNA-seq), could be used to detect signals revealing interactions between host and gut-associated microbial metabolic processes, showing host reliance/utilization of a microbe-derived metabolite. Though not definitive, the corroborative 16 S amplicon marker-based community characterization (and attendant limited predictive-potential functionalities) and CSIA datasets presented here suggest the presence of a gut microbial community in the studied freshwater insects, possibly capable of reducing nitrate to ammonium for host utilization.

Overall, we report higher ^15^N enrichment in the amino acids of the filter-feeding (herbivorous) mayfly (*Hexagenia bilineata*) relative to the predatory dragonfly (*Tetragoneuria spinigera*), potentially attributable to determined differences in microbiome composition (and possibly metabolic capabilities) between the two freshwater insect species. Our results indicate possible host uptake, incorporation, and assimilation of microbe-derived metabolite ^15^NH_4_ into amino acids following microbial ^15^N-nitrate reduction to ammonium (or other processes) occurring in the studied insects. Below, we explore these results and their implications for freshwater insect nutritional ecology, as well as the study’s limitations.

### Variations in Microbiome Structure and Potential Microbial Functional Capabilities

In this study, nymphs from both species were obtained from a supplier - although the specific maintenance regimen was not specified – and kept in the supplied water till the start of the experiment. Acquired nymphs were subsequently incubated in filtered artificial stream water (ASW) without any dietary materials to minimize the introduction of external bacteria during the experiment. Overall, we determined distinct gut microbial community compositions between filter-feeding mayflies and predatory dragonflies at the end of the study. The differences in microbiome composition between the two species, despite the similar experimental conditions and rearing/source environments, may be due to trophic-level effects or selective screening for gut microbial associates. Our results are comparable to previous reports of distinct gut microbiome composition among stream-collected mayflies (grazers, filter-feeders, collector-gatherers) and dragonflies (predators) [21, 22, 25] and possible impacts of abiotic and biotic factors [23, 59]. This suggests the observed differences in community composition and the associated bacteria driving them in this study may mirror differences between the two species in natural systems. Still, this inference will require further verification. Interestingly, we also determined that significant within-species compositional differences between control and treatment groups. This was unexpected, as the control and treatment insects of both species were obtained from the same initial pool and treated the same at every step, except for the inclusion of the heavy (^15^N) or light (_14_N) nitrogen isotope in the added nitrate. The addition of nitrate was not anticipated to alter the composition but to activate microbial nitrate-utilizing processes. However, despite best practices, this within-species difference in community composition may be due to variation among individuals in treatment and control categories, which could be attributed to several factors, such as potential source effects, tank/batch effects, individual heterogeneity, lack of food, and randomization practices. Finally, previous reports also suggest that, to some extent, freshwater insect functional feeding groups (different ecologies) may selectively associate with microbial taxa in their guts that are potentially adaptive to their aquatic niches. This association with potentially adaptive gut microbial associates is well documented in terrestrial insects across various trophic levels [60].

The initial and limited assessment of any adaptive value of the distinct gut microbiomes of both species using FAPROTATX indicated that potential function and bacterial taxa- associations, such as parasite_or_symbionts (attributable to *Anaplasmataceae*, mainly *Wolbachia*) [61], chlorate reducers (attributable to several genera in the family *Rhodocyclaceae* [62], dark hydrogen oxidation (attributable to several genera in the family *Rhodocyclaceae)* [62], denitrification, nitrogen fixation, and nitrogen and nitrite respiration (attributable to several members of the enriched Pseudomonadota families) [63, 64], fermenter and degraders (attributable to several genera in the family *Lachnospiraceae)* [65], and methonotrophs *(*attributable to several genera in the family *Beijerinckiaceae)* [66, 67] were comparatively more present in the predatory dragonflies compared to mayflies (Figs. 3A & B)(Figure S2).

Similarly, several bacterial families that were more differentially abundant in mayflies may be associated with determined differentially abundant potential l functional categories. Potential function and bacterial taxa-associations, such as aromatic hydrocarbon and non-methane hydrocarbon degradation (attributable to several members of the Pseudomonadota families, like *Pseudomonadaceae*) [68–70], sulfate and sulfite respiration pathways [71]; *Pseudomonadaceae* [72, 73]; and *Shewanellaceae* [74], and specifically, nitrate reduction [31, 75–77], *Pseudomonadaceae* [78, 79], *Aeromonadaceae* [58, 71, 80], *Flavobacteriaceae* [81–83], *Shewanellaceae* [31, 84], and *Microbacteriaceae* and *Micrococcaceae* [30, 31, 85] were significantly more enriched in mayflies (Figs. 3A & B)(Figure S1). Overall, since predatory insects consume protein-rich prey, it is likely that the reliance on microbe-mediated biological nitrogen provisioning may be comparatively lower than it is in lower trophic level taxa [14, 17, 86, 87].

### Biological Nitrogen Provisioning Via Nitrate Reduction

Given the present study’s focus on assessing host utilization of microbe-derived ^15^NH_4_ following nitrate reduction to ammonium, we sacrificed the complexity of natural freshwater systems (multiple species per functional feeding group) in favor of a more controlled microcosm study focused on a single species per functional feeding group, with replicates. This approach minimized species variability while enhancing detection of the stable-isotope signal of the hypothesized process likely responsible for nitrogen provisioning within the two species used. Although this limits the generalizability of inferences about differences among species across trophic levels, the study’s primary goal was to examine whether this process occurred in freshwater insects at all. Further studies utilizing multiple species per trophic level would be required before any such generalizable statements can be made about the importance of this process in freshwater insects. The premise of the stable isotope assay is that the ^15^N-tracer signal would be significantly greater in treatment groups than in control groups across both species, by the end of the incubation period (5 days), if the characterized gut microbial associates of both species were carrying out nitrate reduction to ammonium. The 5-day incubation period was selected based on a combination of a previously reported 3-day incubation period [33] and daily monitoring for signs of mortality, especially after the first 3 days. Finally, only insect carcasses (after removal of the digestive system and its contents) were lyophilized and used for the CSIA analysis. This eliminated the microbial contribution to the quantified ^15^N values across amino acids, enabling the detection of insect-only signals.

As anticipated, we detected significantly larger δ^15^N_AA_ signals from hydrolyzed tissues across both non-essential (NEAAs) and essential amino acids (EAAs) in mayflies relative to dragonflies (Figs. 4A & C). The lower δ^15^N_AA_ signal in control mayflies (mayfly_^14^N) compared to treatment mayflies (mayflies_^15^N) confirms the absence of the ^15^N-tracer isotope signal in the control samples, confirming the absence of cross-contamination between mayfly experimental groups. However, the lack of significant signal detection in dragonflies may be attributed to the inability of the associated gut bacteria to reduce nitrate above the baseline background threshold. This could be because they are likely not N-limited and thus not as reliant on microbial nitrogen provisioning. In contrast, the mayflies (*Hexagenia bilineata*; herbivore, filter feeder) used in this study do face considerable nitrogen limitation [15, 17] and possibly rely on a mutualistic BNP function of the associated gut microbiome.

In the mayflies, gut microbial reduction of the supplied K-^15^NO_3_ to ^15^NH_4_ may be followed by host uptake (absorption of ^15^NH_4_ into cells and tissues) and incorporation (routing of ^15^NH_4_ into amino acids and proteins) for biological purposes. Nitrate reduction to ammonium could occur via dissimilatory nitrate reduction to ammonia (DNRA) or assimilatory nitrate reduction to ammonia (ANRA) pathways [30, 31, 76, 79] in mayflies, since we did not control for either process. Within the insect gut, ammonium produced by DNRA is directly available for insect host uptake following bacterial periplasmic nitrate reduction, which reduces nitrate to ammonium outside the bacterial cell without using it for biological purposes within the cytoplasm [30, 31]. Alternatively, the ammonium produced from ANRA within the cytoplasm of gut bacterial cells in the insect gut lumen becomes available to the insect only upon lysis and/or death of bacterial cells within the gut, after the bacterial cells have first used the resulting ammonium for amino acids and peptide biosynthesis [30, 31]. Thus, it is these microbe-derived products (amino acids, peptides, etc.) that become available for uptake by insects from the gut. Evidence of gut microbial nitrate reduction to nitrous oxide (N_2_O) via incomplete denitrification in aquatic macroinvertebrates (including insects) has been previously reported [88–90]. This suggests that some aquatic macroinvertebrate taxa (including freshwater insects) may harbor gut microbial assemblages capable of nitrate reduction (including denitrification), as well as ANRA and DNRA [30, 31], and that these assemblages may alleviate N-limitation. The presence and expression of microbial *nasA* and *nrfA* genes responsible for NRA have been previously reported in the guts of the mayfly (*Ephemera simulans* Walker; family *Ephemeridae* [33], which is closely related to the studied mayfly species in this study (*Hexagenia bilineata*). The comparable microbiome compositions of several mayfly families in the filter feeders and grazers/scrapers functional feeding groups [21], suggest that this microbial function possibly occurs in our study system. Although gene expression data in this case would have been definitive, we focused on CSIA to provide a more direct assessment of this potential function in this study.

A previous study using bulk δ^15^N stable isotope data reported higher ^15^N signals in K-^15^NO_3_ exposed mayflies and caddisflies than in controls [33]. However, 1) the use of bulk δ^15^N data, 2) the inability to show that the provided nitrogen was incorporated into amino acids, and 3) the issue of residual K-^15^NO_3_ on treatment carcass samples before stable isotope analyses, limited any definitive conclusion about the likelihood of this being a function of freshwater insect gut microbiomes. In this study, the experimental design intentionally focused on a single post‑exposure endpoint (5 days) to quantify the steady‑state enrichment of essential and non‑essential amino acids in host tissues, rather than resolving the kinetics of assimilation. Single-endpoint CSIA approaches are commonly used to infer nutrient assimilation and protein synthesis when the system is assumed to have reached an approximate isotopic steady state after a defined labeling period. This approach addressed both issues 1 and 2 of the previous study. We acknowledge that, although the single-endpoint design is appropriate for assessing steady-state enrichment in the study, the absence of time‑series data limits inference about the temporal dynamics of nitrogen flux. We felt it was crucial to assess the possibility of this function before assessing time-series dynamics.

Furthermore, the issue of residual K-^15^NO_3_ on insect carcasses was addressed by keeping the insects in nanopore water 24 h after the end of the study period, then rinsing them once more in water before sample processing. Furthermore, the acidic protein extraction method used by the stable isotope facility (incubating in 6 N HCl for 20 h at 110 °C, followed by solvent extraction) is known to significantly remove non-protein nitrogen (NPN), such as residual nitrate on insect cuticles, thereby minimizing residual effects [91], ensuring that measured values are host-derived rather than environmental (residues on the cuticle).

Additionally, the use of compound-specific (amino acids) isotope analyses addressed concerns 1 and 2 from the previous study, suggesting that ^15^NH_4_ may possibly be incorporated into both essential and non-essential amino acids. Furthermore, we showed that the nitrogen (^15^N) was more detectable in NEAAs than in EAA. This greater routing of microbe-derived ^15^N to NEAAs (which can be synthesized by insects) than to EAAs may be due to insects’ reliance on dietary or microbial sources of EAAs (since they are incapable of synthesizing EAAs). This trend of overall higher ^15^N-signals in NEAAs than in EAAs in insects is attributed to a combination of higher utilization (both synthesis and amino acid turnover) of NEAAs relative to EAAs [92, 93] has been documented in other insects that rely on associated gut bacteria to provide biological nitrogen, such as the Asian longhorned beetle [9] and the pea aphid [92–94]. Although not explicitly tested in this study, extensive literature indicates that the incorporation of biologically derived nitrogen involves the GS/GOGAT pathways [30, 32]. To conclude, our results suggest that gut microbial nitrate reduction could be a route for biological nitrogen provisioning in freshwater insects. However, further experiments are required to confirm this possibility and make causal inferences across different trophic levels, as such information will have implications for the role of gut microbes in aquatic insect trophic ecology and ecosystem-level nitrogen cycling and removal processes.

### Ecosystem Implications of Freshwater Insect Gut Microbial Nitrate Reduction

Several studies have shown that aquatic macroinvertebrates significantly enhance nitrate removal (via N_2_O emissions through incomplete denitrification) from aquatic ecosystems [88–90, 95]. This enhancement is usually attributed to bioturbation and feeding activities of macroinvertebrates in these systems, which exert a top-down effect on nitrate removal by creating microenvironments that enable environmental microbes to reduce nitrate to N_2_O [88, 90, 95]. In these contexts, nitrate removal is not categorically attributed to macroinvertebrate gut microbes but to the physical activities of the macroinvertebrates themselves. Incorporating aquatic macroinvertebrate gut microbial nitrate removal with free-living/environmental microbial nitrate removal processes may 1) potentially add to our understanding of microbial roles in these processes, 2) potentially provide a more comprehensive view of nitrogen cycling in aquatic ecosystems, and **3**) possibly offer new insights into the roles of bacterial symbionts in large-scale nutrient processing. However, the importance of this process may depend on the quality of the freshwater system, as freshwater insects vary in their susceptibility to excess nitrate levels [35, 36]. For example, different mayfly species have varying tolerances to nitrate pollution, with *Heptagenia spp* reported to be highly sensitive to excess nitrate [96–98]. Additionally, dragonflies tend to be more tolerant of nitrate toxicity than mayflies, hence their exclusion from the EPT (Ephemeroptera, Trichoptera, and Plecoptera) taxa index used as bioindicators of stream water quality [99]. Thus, further studies are required to assess the interacting factors underlying differences in freshwater macroinvertebrate gut microbiota, water quality, nitrate concentrations, tolerance limits, and host nitrate-removal abilities.

### Conclusions and Perspectives

In conclusion, we used compound-specific isotope analysis (CSIA) to demonstrate that, as a first proof-of-concept step, biological nitrogen can be made available via gut microbial nitrate reduction in N-limited filter-feeding mayflies more than in predatory dragonflies. This is underscored by distinct gut microbiome composition and potential function between mayflies and dragonflies, which further suggest that this microbial function may be more broadly relevant to N-limited freshwater insects. This suggests a possible symbiotic function of the gut microbiota that alleviates N-limitation, much like gut microbial nitrogen fixation and recycling do in terrestrial insects. However, further studies with rigorous controls are needed to ascertain this gut microbial function in freshwater insects and its generalizability to freshwater insect functional feeding groups.

Although including both bulk and CSIA ^15^N-stable isotope data would have been informative, the cost of analyzing both was prohibitive. Further studies should include isotope data, if possible; if not, preferably CSIA. The utility of CSIA and, crucially, the ability to now quantify and/or visualize stable isotopes in tissues in real time are significant advancements that facilitate the study of freshwater insect-gut microbial symbiotic processes. Additionally, CSIA analyses, coupled with RNA-seq data, could provide a direct assessment of the expressed microbial functional genes *nasA* and *nrfA*, relevant to DNRA and ANRA, respectively, in freshwater insects, rather than the limited assessment of potential functions via FAPROTAX presented here. It was not possible to use both methods in the study due to cost and sample size constraints. However, we believe these approaches may be the best way to further assess this possible microbial function across freshwater insect hosts and, possibly, at the ecosystem level.

## Supplementary Information

Below is the link to the electronic supplementary material.

Supplementary File 1 (DOCX 29.5 KB)

Supplementary File 2 (DOCX 36.0 KB)

## Data Availability

All data supporting the findings of this study are available within the paper and its Supplementary Information. Amplicon data are deposited in GenBank.
