# Supplementary material for: Gut Microbial Nitrate Reduction to Ammonia: A Possible Pathway of Biological Nitrogen Provisioning in Freshwater Insects
Source: Microb Ecol. 2026 Apr 18;89:117. doi: 10.1007/s00248-026-02771-w (PMC13222897; doi:10.1007/s00248-026-02771-w)
Supplement: Supplementary file 1 [file 248_2026_2771_MOESM1_ESM.docx]

**Supplementary Table 1:** Pairwise comparisons of **A**) Shannon diversity index among experimental groups, and **B**) microbial community composition among experimental groups.

| **A. Microbial Evenness Pairwise Comparisons** | **Test-statistic** | **P-value** |  |
| --- | --- | --- | --- |
| Dragonfly_14N vs Mayfly_14N | 0.98 | 0.33 |  |
| Dragonfly_14N vs Dragonfly_15N | -3.21 | <0.05 |  |
| Dragonfly_14N vs Mayfly_15N | 3.37 | <0.05 |  |
| Mayfly_14N vs Dragonfly_15N | -3.76 | <0.05 |  |
| Mayfly_14N vs Mayfly_15N | 1.98 | =0.05 |  |
| Dragonfly_15N vs Mayfly_15N | 6.00 | <0.05 |  |
| **B. Community Composition Pairwise Comparisons** | **F-Value** | **R-Squared** | **P-value** |
| Dragonfly_14N vs Mayfly_14N | 18.56 | 0.32 | <0.00 |
| Dragonfly_14N vs Dragonfly_15N | 4.39 | 0.10 | <0.00 |
| Dragonfly_14N vs Mayfly_15N | 50.09 | 0.54 | <0.00 |
| Mayfly_14N vs Dragonfly_15N | 14.46 | 0.27 | <0.00 |
| Mayfly_14N vs Mayfly_15N | 11.29 | 0.20 | <0.00 |
| Dragonfly_15N vs Mayfly_15N | 38.50 | 0.47 | <0.00 |

**Supplementary Table 2**: **A**) The raw score values of 71 bacterial ASVs (at the family-level) out of 4,389 ASVs (Kruskal–Wallis; FDR-adjusted P-value = 0.05), that differed among the experimental groups following differential abundance analyses, and **B**) The raw score values of 41 differentially abundant potential bacterial metabolic functional groups out of 91 available functional groups following FAPROTAX analysis(Kruskal–Wallis; FDR-adjusted P-value = 0.05), highlighting distinct functional potentials between mayflies and dragonflies.

| **A. Bacterial Families** | **Mayfly_15N** | **Dragonfly_15N** | **Mayfly_14N** | **Dragonfly_14N** |
| --- | --- | --- | --- | --- |
| *A0839* | 2.97 | 0.01 | 1.65 | 0.00 |
| *Abditibacteriaceae* | 0.18 | 0.00 | 0.19 | 0.02 |
| *Aeromonadaceae* | 0.18 | 0.00 | 0.34 | 0.00 |
| *Alcaligenaceae* | 0.16 | 0.01 | 0.02 | 0.00 |
| *Anaerovoracaceae* | 0.13 | 0.00 | 0.12 | 0.00 |
| *Anaplasmataceae* | 0.13 | 0.00 | 0.08 | 0.00 |
| *Bacteroidaceae* | 0.11 | 0.01 | 0.02 | 0.00 |
| *Beijerinckiaceae* | 0.11 | 0.00 | 0.88 | 0.00 |
| *Blastocatellaceae* | 0.08 | 0.05 | 0.00 | 0.00 |
| *Blattabacteriaceae* | 0.07 | 0.00 | 0.02 | 0.00 |
| *Caulobacteraceae* | 0.07 | 0.00 | 0.03 | 0.00 |
| *Chitinibacteraceae* | 0.05 | 0.00 | 1.59 | 0.02 |
| *Chitinophagaceae* | 0.05 | 0.01 | 0.02 | 0.02 |
| *Christensenellaceae* | 0.04 | 0.00 | 0.02 | 0.00 |
| *Clostridia* | 0.00 | 0.02 | 0.00 | 0.00 |
| *Clostridiaceae* | 0.04 | 0.00 | 0.01 | 0.00 |
| *Comamonadaceae* | 0.04 | 0.00 | 0.00 | 0.00 |
| *Deferribacteraceae* | 0.04 | 0.00 | 0.43 | 0.00 |
| *Deinococcaceae* | 0.04 | 0.01 | 0.07 | 0.02 |
| *Desulfobulbaceae* | 0.03 | 0.00 | 0.08 | 0.00 |
| *Desulfovibrionaceae* | 0.03 | 0.00 | 0.02 | 0.00 |
| *Devosiaceae* | 0.03 | 0.00 | 0.00 | 0.00 |
| *Diplorickettsiaceae* | 0.02 | 0.56 | 0.07 | 0.98 |
| *Dysgonomonadaceae* | 0.02 | 0.00 | 0.01 | 0.00 |
| *Enterobacteriaceae* | 0.02 | 0.05 | 0.01 | 0.00 |
| *Erysipelatoclostridiaceae* | 0.02 | 0.46 | 0.03 | 0.15 |
| *Erysipelotrichaceae* | 0.01 | 0.00 | 0.11 | 0.01 |
| *Flavobacteriaceae* | 0.01 | 0.00 | 0.00 | 0.00 |
| *Gallionellaceae* | 0.00 | 0.13 | 0.02 | 0.00 |
| *Gammaproteobacteria* | 0.00 | 0.02 | 0.00 | 0.00 |
| *Hafniaceae* | 0.00 | 0.21 | 0.03 | 0.14 |
| *Hyphomicrobiaceae* | 0.00 | 0.17 | 0.00 | 0.01 |
| *Kaistiaceae* | 0.00 | 0.00 | 0.04 | 0.00 |
| *Lachnospiraceae* | 0.00 | 0.09 | 0.10 | 0.01 |
| *Lactobacillaceae* | 0.00 | 0.57 | 0.00 | 0.77 |
| *Legionellaceae* | 0.00 | 0.12 | 0.00 | 0.01 |
| *Methylophilaceae* | 0.00 | 0.00 | 0.10 | 0.00 |
| *Microbacteriaceae* | 0.00 | 0.32 | 0.02 | 0.07 |
| *Micrococcaceae* | 0.00 | 0.09 | 0.00 | 0.00 |
| *Moraxellaceae* | 0.00 | 0.10 | 0.00 | 0.00 |
| *Muribaculaceae* | 0.00 | 0.06 | 0.01 | 0.00 |
| *Mycoplasmataceae* | 0.00 | 1.18 | 0.00 | 4.71 |
| *Nitrosomonadaceae* | 0.00 | 0.05 | 0.00 | 0.07 |
| *Nocardiaceae* | 0.00 | 0.05 | 0.00 | 0.00 |
| *Oscillospiraceae* | 0.00 | 0.50 | 0.00 | 0.00 |
| *Oxalobacteraceae* | 0.00 | 0.43 | 0.00 | 0.16 |
| *Pirellulaceae* | 0.00 | 1.71 | 0.00 | 1.94 |
| *Prolixibacteraceae* | 0.00 | 0.16 | 0.00 | 0.33 |
| *Pseudomonadaceae* | 0.00 | 0.10 | 0.00 | 0.10 |
| *Rhizobiaceae* | 0.00 | 0.00 | 0.04 | 0.02 |
| *Rhizobiales* | 0.00 | 0.05 | 0.00 | 0.02 |
| *Rhodobacteraceae* | 0.00 | 0.06 | 0.01 | 0.02 |
| *Rhodocyclaceae* | 0.00 | 0.00 | 0.00 | 0.02 |
| *Rikenellaceae* | 0.00 | 0.00 | 0.03 | 0.01 |
| *Rubinisphaeraceae* | 0.00 | 0.11 | 0.00 | 0.01 |
| *Ruminococcaceae* | 0.00 | 0.00 | 0.02 | 0.00 |
| *Shewanellaceae* | 0.00 | 0.02 | 0.00 | 0.00 |
| *Sphingobacteriaceae* | 0.00 | 0.02 | 0.00 | 0.00 |
| *Sphingomonadaceae* | 0.00 | 0.02 | 0.00 | 0.00 |
| *Spirosomaceae* | 0.00 | 0.00 | 0.01 | 0.00 |
| *Sporomusaceae* | 0.00 | 0.07 | 0.00 | 0.00 |
| *Streptococcaceae* | 0.00 | 0.03 | 0.00 | 0.00 |
| *Sutterellaceae* | 0.00 | 0.12 | 0.00 | 0.00 |
| *T34* | 0.00 | 0.03 | 0.00 | 0.00 |
| *Tannerellaceae* | 0.00 | 0.03 | 0.00 | 0.00 |
| *Unknown* | 0.00 | 0.09 | 0.00 | 0.00 |
| *vadinBB60* | 0.00 | 0.11 | 0.00 | 0.00 |
| *vadinBB60* | 0.00 | 0.02 | 0.00 | 0.00 |
| *Weeksellaceae* | 0.00 | 0.03 | 0.00 | 0.00 |
| *Xanthobacteraceae* | 0.00 | 0.02 | 0.00 | 0.00 |
| *Xanthomonadaceae* | 0.00 | 0.13 | 0.00 | 0.00 |
| *Yersiniaceae* | 0.00 | 0.03 | 0.00 | 0.00 |
| **B. Potential Microbial Functional Groups** | **Dragonfly_14N** | **Dragonfly_15N** | **Mayfly_14N** | **Mayfly_15N** |
| ureolysis | 3.32 | 5.63 | 0.07 | 0.09 |
| sulfate_respiration | 0.00 | 0.01 | 0.16 | 4.13 |
| respiration_of_sulfur_compounds | 0.00 | 0.01 | 0.16 | 4.13 |
| intracellular_parasites | 42.01 | 13.81 | 0.01 | 0.27 |
| aerobic_ammonia_oxidation | 0.01 | 0.06 | 0.00 | 0.00 |
| dark_oxidation_of_sulfur_compounds | 0.07 | 0.61 | 0.01 | 0.23 |
| human_pathogens_all | 0.01 | 0.10 | 0.00 | 0.01 |
| human_associated | 0.20 | 2.62 | 0.00 | 0.04 |
| nitrification | 0.10 | 0.06 | 0.00 | 0.00 |
| hydrocarbon_degradation | 0.01 | 0.08 | 0.16 | 1.40 |
| animal_parasites_or_symbionts | 0.20 | 2.63 | 0.00 | 0.04 |
| phototrophy | 2.36 | 1.49 | 0.18 | 0.28 |
| photoautotrophy | 0.00 | 0.04 | 0.00 | 0.00 |
| photoheterotrophy | 2.36 | 1.45 | 0.18 | 0.28 |
| nitrogen_fixation | 0.00 | 0.09 | 0.00 | 0.02 |
| methanotrophy | 0.00 | 0.02 | 0.06 | 0.21 |
| aromatic_hydrocarbon_degradation | 0.01 | 0.06 | 0.10 | 1.19 |
| aliphatic_non_methane_hydrocarbon_degradation | 0.01 | 0.06 | 0.10 | 1.19 |
| anoxygenic_photoautotrophy_S_oxidizing | 0.00 | 0.02 | 0.00 | 0.00 |
| anoxygenic_photoautotrophy | 0.00 | 0.02 | 0.00 | 0.00 |
| aerobic_nitrite_oxidation | 0.08 | 0.01 | 0.00 | 0.00 |
| chlorate_reducers | 0.46 | 0.10 | 0.01 | 0.01 |
| human_gut | 0.19 | 2.52 | 0.00 | 0.03 |
| mammal_gut | 0.19 | 2.52 | 0.00 | 0.03 |
| methanol_oxidation | 0.41 | 0.82 | 0.02 | 0.10 |
| aromatic_compound_degradation | 0.21 | 1.08 | 0.49 | 1.52 |
| xylanolysis | 0.00 | 0.10 | 0.00 | 0.00 |
| sulfite_respiration | 0.00 | 0.00 | 0.03 | 0.50 |
| photosynthetic_cyanobacteria | 0.00 | 0.02 | 0.00 | 0.00 |
| oxygenic_photoautotrophy | 0.00 | 0.02 | 0.00 | 0.00 |
| predatory_or_exoparasitic | 0.12 | 0.07 | 0.04 | 0.01 |
| nitrate_denitrification | 0.28 | 0.71 | 0.01 | 0.07 |
| nitrite_denitrification | 0.28 | 0.71 | 0.01 | 0.07 |
| denitrification | 0.28 | 0.71 | 0.01 | 0.07 |
| nitrite_respiration | 0.28 | 0.71 | 0.01 | 0.07 |
| nitrate_respiration | 1.29 | 1.48 | 1.85 | 1.67 |
| nitrogen_respiration | 1.29 | 1.48 | 1.85 | 1.67 |
| dark_hydrogen_oxidation | 0.39 | 0.21 | 0.06 | 0.08 |
| chemoheterotrophy | 38.20 | 39.99 | 39.41 | 60.29 |
| **nitrate_reduction** | **1.98** | **1.67** | **28.29** | **9.78** |
| fermentation | 3.37 | 16.21 | 26.74 | 10.53 |
