# Supplementary material for: Gut Microbial Nitrate Reduction to Ammonia: A Possible Pathway of Biological Nitrogen Provisioning in Freshwater Insects
Source: Microb Ecol. 2026 Apr 18;89:117. doi: 10.1007/s00248-026-02771-w (PMC13222897; doi:10.1007/s00248-026-02771-w)
Supplement: Supplementary file 2 [file 248_2026_2771_MOESM2_ESM.docx]

**Supplementary Figure 1**. The rarefaction curve across the four experimental groups shows that the rarefaction depth (5,798 reads per sample) adequately captured diversity among samples.

**Supplementary Figure 2**. The relative abundances of 137differentially abundant bacterial ASVs (at the genus level) (Kruskal–Wallis; FDR-adjusted P-value = 0.05), likely driving differences in determined microbial community composition between mayflies and dragonflies.
